# Resampling a carrion beetle fauna after 40 years (Coleoptera, Staphylinidae, Silphinae, and Leiodidae, Cholevinae)

**DOI:** 10.3897/BDJ.13.e151206

**Published:** 2025-03-31

**Authors:** Menno Schilthuizen, Teun van der Sterren, Isabel Kersten, Mike Groenhof, Henk van der Meulen, Leonie Wezendonk

**Affiliations:** 1 Taxon Expeditions B.V., Leiden, Netherlands Taxon Expeditions B.V. Leiden Netherlands; 2 Leiden University, Leiden, Netherlands Leiden University Leiden Netherlands; 3 Taxon Foundation, Leiden, Netherlands Taxon Foundation Leiden Netherlands; 4 Naturalis Biodiversity Center, Leiden, Netherlands Naturalis Biodiversity Center Leiden Netherlands; 5 Thomas a Kempis College, Arnhem, Netherlands Thomas a Kempis College Arnhem Netherlands; 6 Institute for Biology Leiden, Leiden, Netherlands Institute for Biology Leiden Leiden Netherlands; 7 GELIFES, Groningen University, Groningen, Netherlands GELIFES, Groningen University Groningen Netherlands

**Keywords:** carrion trapping, insect declines, Staphylinidae, Leiodidae, education

## Abstract

**Background:**

From 29 May until 5 June 1982, the first author placed baited pitfall traps to sample the Staphylinidae, Silphinae and Leiodidae, Cholevinae (Coleoptera) fauna in a mixed forest in the Netherlands. Exactly 40 years later (29 May until 5 June 2022), as a project on insect declines with high school students at a nearby school, a resampling was carried out.

**New information:**

We report the silphine and cholevine specimens recorded both in 1982 and 2022. We found that total species richness and biomass had not noticeably changed, but there were some distinct appearances and disappearances of species. In addition, the species abundance distribution has become more skewed in favour of locally common species, such as *Sciodrepoideswatsoni* and *Fissocatopswesti*. We discuss our results in the context of insect declines worldwide.

## Introduction

In recent years, many studies have appeared that investigate the rate of decline in insect densities and diversities (e.g. Hallmann et al. (2017), Van Klink et al. (2020)). These studies essentially depend on the availability of long-term surveys in the same area using standardised sampling techniques. While studies with temporally dense sampling (continuous, annual or with regular longer intervals) are obviously most valuable, comparisons between two time-points (e.g. Cuesta and Lobo (2019), Opito et al. (2023)) can also add important data to the study of long-term trends in entomofauna.

In 1982, the first author (M.S.), as high school student and encouraged by his biology teacher, Daan Vestergaard, conducted a one-week inventory of carrion-visiting beetles (Silphinae and Cholevinae) with baited pitfall traps in the Lichtenbeek Estate near Oosterbeek, the Netherlands. In this paper, we describe the results of a resampling, using exactly the same inventory technique, precisely 40 years later by a student-teacher team from a nearby school, in the context of understanding environmental change and insect declines.

Our study showed no noticeable differences in species richness, total abundance and biomass between the two sampling years. However, in 2022, the species abundance distribution had a lower evenness and a more hollow shape than in 1982: a few very abundant species, many very rare species and very few species with intermediate abundances. Furthermore, there are several striking differences in the abundance of specific species, most of which are species that have declined compared to 1982, but also some that have increased.

For some species that showed conspicuous changes in abundance, it is possible to speculate on the causes. For example, *Nicrophorushumator* is a common forest species with two discrete abundance peaks, in April-May and in August (Schilthuizen and Vallenduuk 1998, Colijn and Heijerman 2020). The fact that we found 130 individuals in 1982 and only four in 2022 could be the result of a shift in the phenology. The biological onset of spring has advanced by over two weeks in the past few decades in the Netherlands (Van Vliet et al. 2014), so it is possible that the 1982 sampling fell within the spring peak of *N.humator*, whereas during the 2022 sampling, the peak was already over. For other species, for example, *Oiceoptomathoracicum*, which has been more frequently reported in the past decades and *Catopssubfuscus*, for which records appear to have declined (Schilthuizen, pers. obs.), the changes may reflect true changes in the nationwide population density of these species.

The data we present describe a comparison in Silphinae and Cholevinae communities at two time points exactly 40 years apart. While the limited scope of the comparison precludes any far-reaching interpretation, the patterns we found (no declines in species richness, total abundance and biomass, but distinctly reduced evenness) might point to a general trend and the data could be used for ongoing meta-analyses on insect declines. A note of caution is, however, warranted, because of changes in the sampling set-up (for example, ethanol vs. salt water as preservative), the weather and the habitat.

## Project description

### Title

Resampling the carrion beetles of Lichtenbeek Estate 40 years later

### Study area description

The 89 ha Lichtenbeek Estate (52.00°N 05.84°E) is a mixed forest near Arnhem, the Netherlands. It is located partly within the Municipality of Arnhem, partly in that of Renkum. On the north, it is bordered by the N224 provincial road, on the west by a smaller road, the Dreijenseweg. On the east and southeast, the forest is adjoined by pasture and arable land and on the south by a residential area in the northern part of the town of Oosterbeek. We also included the western portion of the smaller forest of Vijverberg, located to the north of the N224, which holds the springs that used to feed the brook after which Lichtenbeek was named. The forest is located at ca. 50 m elevation on the south-exposed slope of the glacial moraine north of the Rhine and has a podzol soil of fine to coarse sand. Its vegetation is composed of separated stands, some of which contain primarily deciduous trees (*Fagussylvatica*, *Quercus* sp.), others coniferous trees (e.g. *Pseudotsugamenziesii*). As no major changes have taken place in the layout or tree vegetation of the forest over the past 40 years, we cannot rule out that, although some plots may have been logged and regrown, others may have matured. Permission to conduct this study was granted by Stichting Geldersch Landschap & Kastelen (through Mr. Bram de Jong).

### Design description

**Figure 1.**
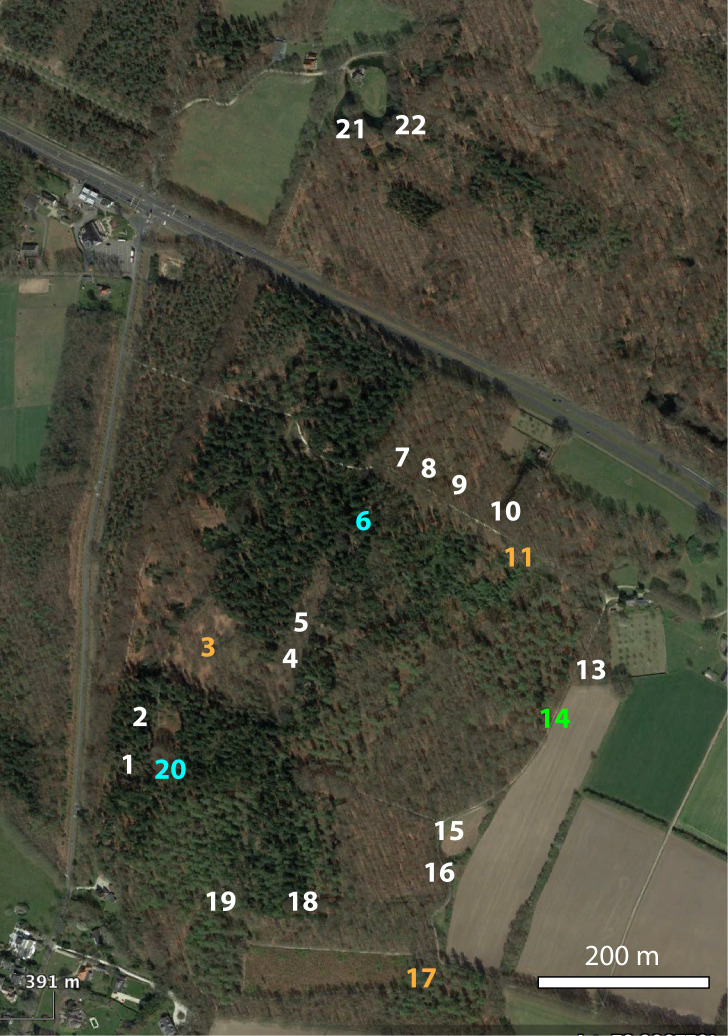
Map of the Lichtenbeek Estate (satellite image provided by Google Earth) and the positions of the traps. Colours of the numbers indicate the bait type used: white, meat; orange, cheese; blue, mushrooms; green, apple.

In the week of 29.v. - 5.vi.1982, one of us (M.S.) used 1 litre cans with a 10 cm diameter opening as pitfall traps. These were dug in so that the top was flush with the soil surface and baited with 100 g each of horse meat (16 traps), mushrooms (2 traps), Dutch old cheese (3 traps) and apple (1 trap); see Fig. 1 for a map of the trap positions. A square of chicken wire was placed over each trap and secured with tent pegs; from the chicken wire, the bait was suspended. As preservative in the traps, 70% ethanol was used. The traps were emptied once, at the end of the sampling week and were then removed. All collected material of Silphinae and Cholevinae was identified by M.S. to species level, where necessary using genital dissection and is currently in the collection of Naturalis Biodiversity Center, Leiden, the Netherlands; small numbers of duplicates are retained in the collection of Taxon Foundation, Leiden, the Netherlands. The data were reported on in a different context in Schilthuizen (2022). The local weather during the 1982 sampling week (at weather station Deelen, 8 km NNE of Lichtenbeek; obtained from https://daggegevens.knmi.nl) was very warm (daily mean temperature 20.5°C), very little wind (daily mean wind speed 2.9 m/s), sunny (daily mean sunshine duration 11.4 h) and mostly dry (two days with rain, 0.3 and 1.7 mm, respectively).

In the week of 29.v. – 5.vi.2022, using 1982 sketch maps and notes kept by M.S., we replicated the trapping procedure of 1982, with the following exceptions: we used 1 litre plastic yoghurt tubs with, as preservative, a saturated sodium chloride (NaCl) solution and a drop of dishwashing detergent; horse meat was replaced by beef. One trap (No. 12) was lost, so we removed the catch from this trap also from the 1982 dataset. All collected material of Silphinae was sorted by T.v.d.S. and I.K., identified by T.v.d.S and checked by M.S. The Cholevinae were sorted by T.v.d.S., I.K. and M.S. and identified by M.S. One badly damaged female *Choleva* could not be identified to species. All specimens were deposited into the collection of Taxon Foundation, Leiden, the Netherlands, except for a small number of Silphinae duplicates (two specimens per species), which were kept in the private collection of T.v.d.S. The local weather (Deelen data) during the 2022 sampling week was cooler than in 1982 (daily mean temperature 13.7°C), there was also very little wind (daily mean wind speed 3.3 m/s), it was less sunny (daily mean sunshine duration 6.3 h) and wetter (four days with rain, 0.9, 2.0, 2.6 and 27.1 mm, respectively).

All specimens of all Silphinae and Cholevinae species were counted, tabulated and compared in PAST 4.11 (Hammer et al. 2001). To estimate biomass, based on Schilthuizen and Vallenduuk (1998) and an assumed specific weight similar to that of water, we used an average volume of Silphinae of 3 x 4 x 12 mm = 144 mg and of Cholevinae of 1.5 x 2 x 4 mm = 12 mg.

In 1982 and 2022, we collected 1151 and 1523 individuals, respectively, from a total of nine Silphinae and 16 Cholevinae species. Not all species were found in both years. In 1982 (21 species found), one silphine (*Silphatristis*) and three cholevines (*Catopschrysomeloides*, *Catopskirbyi* and *Catopssubfuscus*) were found that were not found in 2022 and, in 2022 (20 species found), two silphines (*Silphaobscura* and *Oiceoptomathoracicum*) and three cholevines (*Catopsneglectus*, *Choleva* sp., and *Ptomaphagussubvillosus*) were found that were not found in 1982. Margalef’s species diversity index (Clifford and Stephenson 1975) was 2.696 in 1982 and 2.729 in 2022 and Buzas & Gibson’s evenness (Buzas and Gibson 1969) was 0.444 and 0.213 in 1982 and 2022, respectively. The estimated biomass was 86.412 g and 82.824 g in 1982 and 2022, respectively.

**Figure 2.**
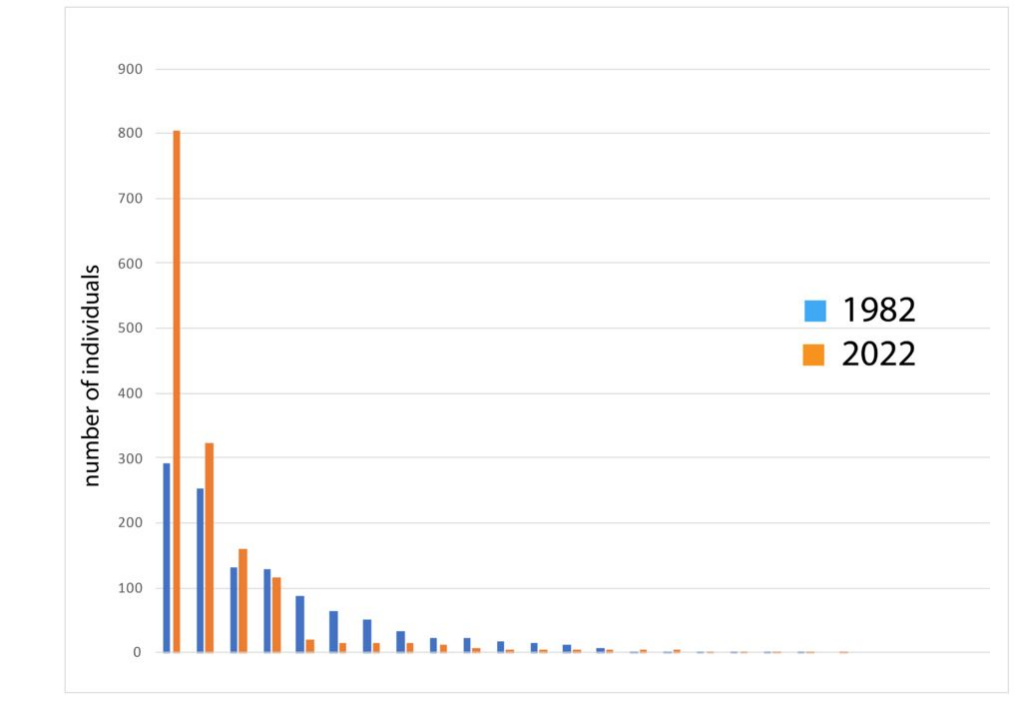
Species abundance distributions, organised from most common on the left to rarest on the right, across all traps and all species for both sampling periods (one species per column).

All data have been uploaded to GBIF, and summarised in Fig. 2 and Tables 1, 2. We found a significant difference in species composition between the two sampling years (Chi-squared = 815.87, d.f. = 24, p < 0.000001). In particular, strong differences of an order of magnitude or more were found in the abundances of *Nicrophorushumator*, *Silphatristis*, *Oiceoptomathoracicum*, *Catopspicipes*, *Catopssubfuscus*, *Catopstristis*, *Fissocatopswesti*, *Nargusvelox* and *Ptomaphagusmedius*. Of these species, six declined and three rose in abundance (see Table 1).

### Funding

This study was supported by the Prins Bernhard Cultuurfonds (Gelderland) and the Suzanne Hovinga Stichting.

## Geographic coverage

### Description

The study was conducted on the Lichtenbeek Estate near Oosterbeek, Province of Gelderland, the Netherlands.

### Coordinates

51.9978 and 52.0065 Latitude; 5.844 and 5.8551 Longitude.

## Taxonomic coverage

### Description

Coleoptera, Staphylinidae, Silphinae (carrion beetles).

Coleoptera, Leiodidae, Cholevinae (round fungus beetles).

### Taxa included

**Table:** 

Rank	Scientific Name	Common Name
subfamily	Cholevinae	round fungus beetles
subfamily	Silphinae	carrion beetles

## Temporal coverage

**Data range:** 1982-5-29 – 2022-6-05.

## Collection data

### Collection name

Taxon Expeditions

### Collection identifier

TXEX

### Specimen preservation method

dry and in ethanol

## Usage licence

### Usage licence

Creative Commons Public Domain Waiver (CC-Zero)

## Data resources

### Data package title

Carrion beetles Lichtenbeek

### Resource link

https://ipt.pensoft.net/resource?r=carrionbeetleslichtenbeekevents

### Number of data sets

2

### Data set 1.

#### Data set name

Data Resources 1 (sampling events)

#### Description

This dataset describes the sampling events with baited pitfall traps in Lichtenbeek Estate, Oosterbeek, the Netherlands, on two occasions: 1982 and 2022. These have been uploaded to GBIF.

**Data set 1.** 

Column label	Column description
eventID	Code for each pitfall collection event.
samplingProtocol	Type of sampling method.
samplingEffort	The duration of the sampling.
sampleSizeValue	The value of the trap volume.
sampleSizeUnit	The unit of measurement for sampling size.
eventDate	The time-span covered by the sampling event.
country	The country where the sampling event took place.
countryCode	The code for the country where the sampling event took place.
locality	The name of the locality where the sampling event took place.
locationID	An identification code for the location of the sampling event.
decimalLatitude	The latitude of the location of the sampling event in decimal degrees.
decimalLongitude	The longitude of the location of the sampling event in decimal degrees.
geodeticDatum	The geodetic datum for the longitude and latitude locator used.
coordinateUncertaintyInMetres	The uncertainty in metres about the coordinates.
type	type
ownerInstitutionCode	The institution where the samples are held.

### Data set 2.

#### Data set name

Data Resources 2 (occurrences)

#### Description

Specimens recorded for each sampling event.

**Data set 2.** 

Column label	Column description
eventID	Code for each pitfall collection event.
occurrenceID	Code for each set of specimens recorded per sampling event.
basisOfRecord	The basis on which the record was made.
organismQuantity	The number of individuals aggregated into the record.
organismQuantityType	Qualifier for the types of organisms recorded.
occurrenceStatus	Present or absent.
scientificName	The scientific name of the taxon recorded.
kingdom	The kingdom to which the taxon belongs.
phylum	The phylum to which the taxon belongs.
class	The class to which the taxon belongs.
order	The order to which the taxon belongs.
family	The family to which the taxon belongs.
taxonRank	The taxonomic rank of the taxon.
identifiedBy	The name of the authority responsible for the identification.
recordedBy	The organisation responsible for the record.
type	type of record.
ownerInstitutionCode	The organisation or person owning the data.

## Figures and Tables

**Table 1.** Numbers of individuals found for all species, pooled per sampling period for all traps.

**Family**	**Species**	**N (1982)**	**N (2022)**
Staphylinidae	*Nicrophorushumator*	130	4
Staphylinidae	*Nicrophorusvespillo*	18	21
Staphylinidae	*Nicrophorusvespilloides*	254	117
Staphylinidae	*Phosphugaatrata*	1	4
Staphylinidae	*Silphacarinata*	132	322
Staphylinidae	*Silphaobscura*	0	2
Staphylinidae	*Silphatristis*	13	0
Staphylinidae	*Oiceoptomathoracicum*	0	14
Staphylinidae	*Thanatophilussinuatus*	2	5
Leiodidae	*Catopschrysomeloides*	1	0
Leiodidae	*Catopscoracinus*	88	13
Leiodidae	*Catopsfuliginosus*	6	5
Leiodidae	*Catopskirbyi*	1	0
Leiodidae	*Catopsneglectus*	0	4
Leiodidae	*Catopsnigricans*	1	3
Leiodidae	*Catopspicipes*	34	5
Leiodidae	*Catopssubfuscus*	24	0
Leiodidae	*Catopstristis*	65	6
Leiodidae	*Choleva* sp.	0	1
Leiodidae	*Fissocatopswesti*	15	161
Leiodidae	*Nargusvelox*	23	1
Leiodidae	*Ptomaphagusmedius*	1	15
Leiodidae	*Ptomaphagussubvillosus*	0	1
Leiodidae	*Sciodrepoidesfumatus*	50	15
Leiodidae	*Sciodrepoideswatsoni*	292	804
	**total**	**1151**	**1523**

**Table 2.** Summary statistics for the two sampling periods.

	**1982**	**2022**
**Species richness**	21	20
**Number of specimens**	1151	1523
**Margalef's diversity**	2.696	2.729
**Biomass (g)**	86.412	82.824
**Evenness (95% C.I.)** **Shannon's H** **1/D (Simpson)**	0.444 (0.432 - 0.551) 2.184 6.562	0.213 (0.204 - 0.252) 1.501 2.936
